# The Evolution of a Female Genital Trait Widely Distributed in the Lepidoptera: Comparative Evidence for an Effect of Sexual Coevolution

**DOI:** 10.1371/journal.pone.0022642

**Published:** 2011-08-17

**Authors:** Víctor Sánchez, Blanca Estela Hernández-Baños, Carlos Cordero

**Affiliations:** 1 Posgrado en Ciencias Biológicas, Instituto de Ecología, Universidad Nacional Autónoma de México, Mexico City, Distrito Federal, Mexico; 2 Departamento de Biología Evolutiva, Facultad de Ciencias, Universidad Nacional Autónoma de México, Mexico City, Distrito Federal, Mexico; 3 Instituto de Ecología, Universidad Nacional Autónoma de México, Mexico City, Distrito Federal, Mexico; The University of Queensland, St. Lucia, Australia

## Abstract

**Background:**

Sexual coevolution is considered responsible for the evolution of many male genital traits, but its effect on female genital morphology is poorly understood. In many lepidopterans, females become temporarily unreceptive after mating and the length of this refractory period is inversely related to the amount of spermatophore remaining in their genital tracts. Sperm competition can select for males that delay female remating by transferring spermatophores with thick spermatophore envelopes that take more time to be broken. These envelopes could select for signa, sclerotized sharp structures located within the female genital tract, that are used for breaking spermatophores. Thus, this hypothesis predicts that thick spermatophore envelopes and signa evolve in polyandrous species, and that these adaptations are lost when monandry evolves subsequently. Here we test the expected associations between female mating pattern and presence/absence of signa, and review the scant information available on the thickness of spermatophore envelopes.

**Methodology/Principal Findings:**

We made a literature review and found information on female mating pattern (monandry/polyandry), presence/absence of signa and phylogenetic position for 37 taxa. We built a phylogenetic supertree for these taxa, mapped both traits on it, and tested for the predicted association by using Pagel's test for correlated evolution. We found that, as predicted by our hypothesis, monandry evolved eight times and in five of them signa were lost; preliminary evidence suggests that at least in two of the three exceptions males imposed monandry on females by means of specially thick spermatophore envelopes. Previously published data on six genera of Papilionidae is in agreement with the predicted associations between mating pattern and the characteristics of spermatophore envelopes and signa.

**Conclusions/Significance:**

Our results support the hypothesis that signa are a product of sexually antagonistic coevolution with spermatophore envelopes.

## Introduction

The convergence and divergence of male and female interests during sexual interactions generates reciprocal selection pressures that can result in the development of male and female co-adaptations, a process known as sexual coevolution [1]–[4]. Depending on the nature of the selective pressures, sexual coevolution is driven by mate choice [2], [5], sexual conflict (the so-called “sexually antagonistic coevolution”; [4], [6]) or a mixture of both [7]. Empirical evidence supports the hypothesis that sexual coevolution is responsible for the evolution of many male genital traits ([2], [3], [6], [8]–[12], but see [13]). As predicted by this hypothesis, in general male genitalia are complex organs that evolve rapidly and divergently [2], [8], [10]. However, the fact that female genitalia are morphologically simpler and uniform in several taxa [2], [14] is somewhat paradoxical since sexual coevolution predicts evolutionary responses in both sexes. It can be argued that evolutionary responses in females are more difficult to detect because they occur at the level of the nervous and endocrine systems [2], [3], [9], whereas male adaptations involve morphological modifications. However, recent studies indicate that in some groups female morphological adaptations also have evolved [11], [12].

**Figure 1:**
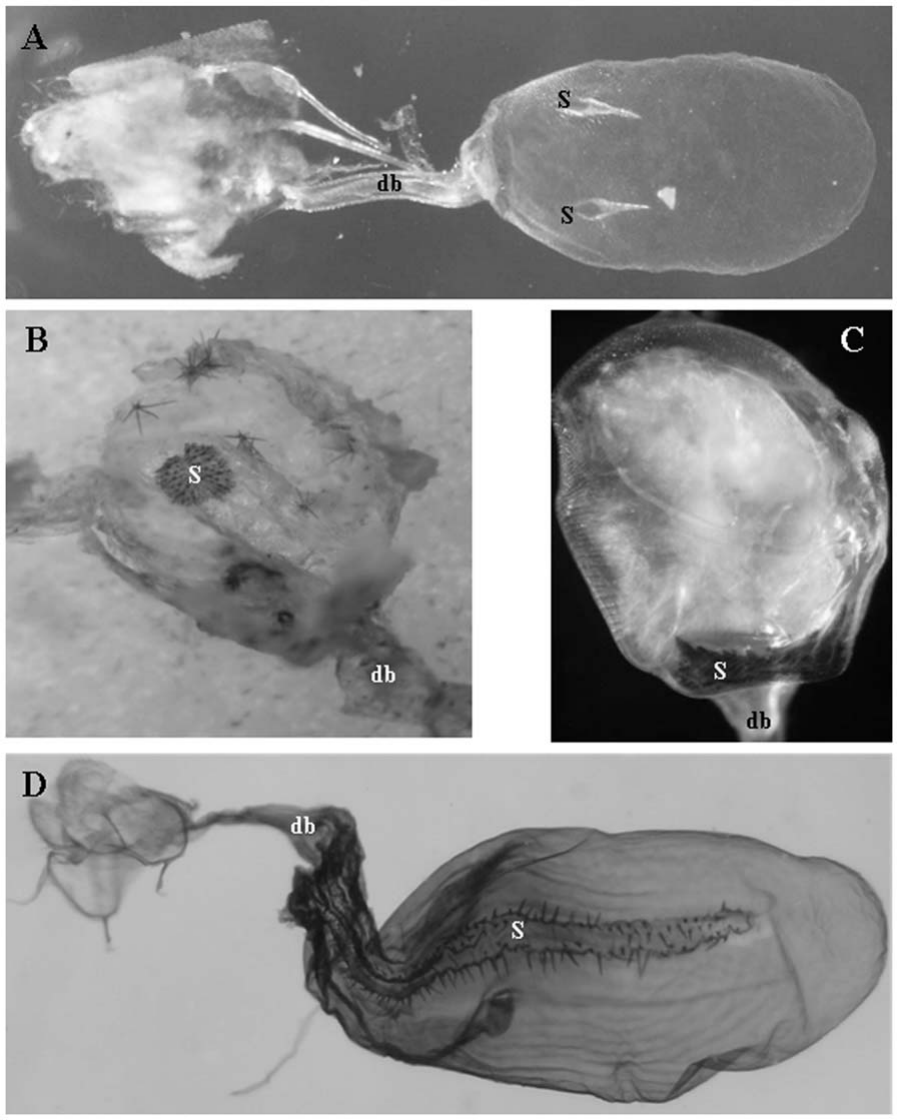
A sampler of the morphological diversity of signa in female Lepidoptera. Each signum is indicated by an “S”. (A) *Callophrys xami* (Lycaenidae): signa are a pair of thin thorns. (B) *Erbessa priverna*: (Notodontidae): signum is a plate covered by small thorns. (C) *Pyrisitia nise* (Pieridae): signum is a strong structure covered by thick spines of different lengths. (D) *Ephialtias draconis* (Notodontidae): signum is a long, narrow, concave structure with thin spines along the margins of its internal surface. In (A), (C) and (D) the signa are observed through the wall of the corpus bursae, whereas in (B) the corpus bursae was opened and two spermatophores removed. In (B) several deciduous cornuti shed from the male endophallus are attached to the corpus bursae wall, and in (C) there are spermatophore remains within the corpus bursae. db: ductus bursae. Photographs are at different scales.

**Figure 2:**
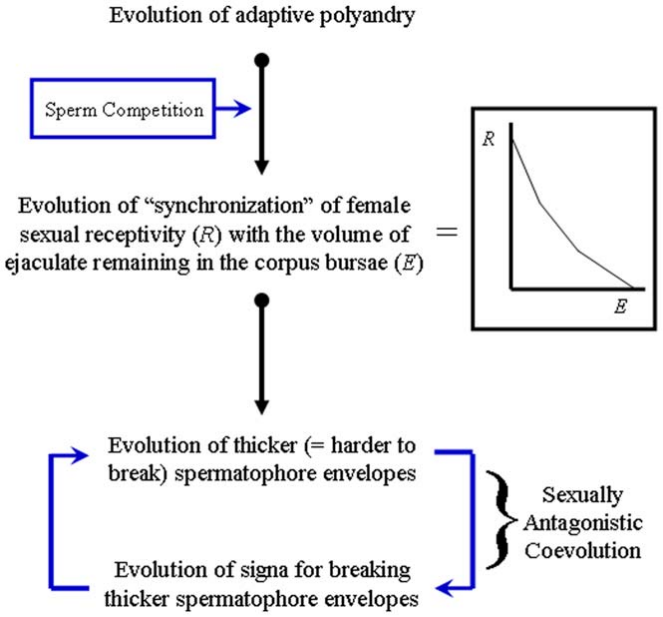
Schematic depiction of the sexually antagonistic coevolution hypothesis for the evolution of signa. Black arrows represent evolutionary transitions and blue arrows selective pressures.

Here, we present evidence supporting a sexually antagonistic coevolution hypothesis for the evolution of female genital sclerotized structures called signa, present in many species of Lepidoptera [15]. Signa are located on the inner wall of the corpus bursa, a sac-like organ in which males deposit a spermatophore during copulation (Figure 1), and their main function is to break off the external wall of the spermatophore, thus allowing females access to the resources contained in it [16], [17]. Our hypothesis proposes the following sequence of evolutionary steps (Figure 2) [15]: (1) Polyandry evolves, possibly to increase the acquisition of resources contained in spermatophores such as nutrients, hormone-like substances, etc. [18]–[22]. Available data indicates that polyandry is widespread in Lepidoptera [2], [21], [23] and the (also widespread) taxonomic distribution of polyandry in insects [2], [24] suggests that this mating pattern could be plesiomorphic in Lepidoptera. (2) Polyandrous females evolve an inverse relationship between their sexual receptivity and the amount of spermatophore remaining in their corpus bursa to optimize the balance between replenishment of sperm and spermatophore resources and remating costs (such as decreased time for foraging and egg laying, predation risk, etc.). This results in a positive relationship between amount of spermatophore transferred and length of the period of female sexual refractoriness. The expected correlations exist in several polyandrous Lepidoptera [15], [23], [25]. (3) Sperm competition generated by polyandry selects for males that produce spermatophore envelopes more difficult to break, thus increasing the lengths of female refractory period and time to remating [15], [23]. (4) Since the optimal female refractory period is expected to be shorter for females than for their mates (for example, females may remate to replenish spermatophore resources or to “renew” sperm stores when they still have viable sperm from the previous male), spermatophore envelopes difficult to break favor the evolution of signa as female devices that increase the rate at which envelopes are torn open, thus moving the rate of recovery of sexual receptivity back to the female's optimum. The process described in (3) and (4) could continue through time (Figure 2) [15]. Therefore, this hypothesis predicts that (a) signa evolve in polyandrous species, and that (b) if subsequently monandry evolves (either because it is selected for in females, or because males evolve alternative adaptations to induce monandry such as mating plugs that render thick spermatophore envelopes redundant), selection will favor thinner/easier to break spermatophore envelopes that reduce costs of spermatophore production, which, in turn, (c) will favor the reduction/loss of signa. Here, we test predictions (a) and (c) by means of a comparative phylogenetic analysis.

## Methods

We collected published data on signa (presence or absence) and female mating pattern (monandry or polyandry) for 37 taxa (Table 1). Species names were actualized according to information in www.nic.funet.fi/pub/sci/bio/life/intro.html (consulted 5/27/2011); a table with the names used in the original references can be obtained from the corresponding author. We employed the Matrix Representation Using Parsimony method [26]–[28] to obtain phylogenetic “supertrees” for these taxa from seven partial source phylogenies in which the branches relevant to this study are well supported (see references in Table 1). We mapped female mating pattern and presence/absence of signa in the most parsimonious and the consensus supertrees, and looked for correlated evolution between these traits by using Pagel's test for correlated evolution [29]. Pagel's test compares a model of correlated evolution with a model of independent evolution of the two traits using maximum likelihood. This test is in *BayesDiscrete* module of the *BayesTraits* software developed by Pagel and Meade (http://www.evolution.rdg.ac.uk/BayesTraits.html). To apply Pagel's test to the consensus tree it was necessary to collapse the polytomy including *Phoebis*, *Colias* and *Gonepterix*, reducing our sample to 35 taxa.

**Table 1:** Sources of data on signa (presence or absence), female mating pattern (polyandry or monandry) and phylogeny, used in the comparative phylogenetic analysis summarized in Figure 3.

	References		
Species	Signa	Mating Pattern	Phylogeny
*Nepticula macrocarpae*	[34]		[61]
Peridroma saucia*	[35]	[23]	[62]
Spodoptera ochrea*	[36]	[21], [23]	[62]
Hadula trifolii*	[37]	[53]	[62]
Urbanus proteus proteus*, U. acawoios, U. belli, U. dubius, U. elmina, U. esma, U. esmeraldus, U. esta, U. evona, U. huancavillcas, U. magnus, U. prodicus, U. pronta, U. pronus, U. viridis, U. viterboana	[38]	[23]	[61]
Parnassius glacialis*, P. stubbendorfi*	[33]	[33]	[63]
Luehdorfia japonica*, L. puziloi*	[33]	[33]	[63]
Graphium meeki inexpectatum*, G. doson*, G. sarpedon*	[33]	[33]	[63]
Atrophaneura alcinous*	[33]	[33]	[63]
Pachliopta aristolochiae*	[33]	[33]	[63]
Papilio bianor dehaani*, P. helenus*, P. junia*, P. maackii*, P. macilentus*, P. machaon*, P. memnon*, P. okinawaensis*, P. polytes*, P. protenor*, P. xuthus*	[33]	[33]	[63]
Aporia crataegi*	[39]	[54]	[64]
Pieris brassicae*, P. napi*, P. rapae*, P. beckeri	[40]–[42]	[23], [54], [55]	[64]
Pontia daplidice*, P. protodice*, P. callidice, P. occidentalis, P. sisymbrii	[41]	[50]	[64]
Phoebis sennae*, P. editha	[43]	[23]	[64]
Gonepteryx rhamni*	[39]	[54], [55]	[64]
Colias philodice*	Pers. obs.	[23]	[64]
*Heliconius 1* H. astraea*, H. atthis*, H. besckei*, H. burneyi*, H. cydno*, H. egeria*, H. elevatus*, H. ethilla*, H. hecale*, H. heurippa*, H. ismenius*, H. luciana*, H. melpomene*, H. nattereri*, H. numata*, H. pardalinus*, H. timareta*, H. wallacei*,	[44]	[23], [56]	[58], [65]
*Heliconius 2* H. xanthocles*, H. clysonymus*, H. congener*, H. charitonia*, H. demeter*, H. eleuchia*, H. erato*, H. hecalesia*, H. hermathena*, H. hewitsoni*, H. hortense*, H. leucadia*, H. ricini*, H. sapho*, H. sara*, H. telesiphe*	[44]	[23], [56]	[58], [65]
Laparus doris*	[44]	[44]	[66]
*Eueides 1* E. aliphera*, E. heliconioides*, E. lybia*, E. tales*	[44]	[57]	[58]
*Eueides 2* E. emsleyi*, E. isabella*, E. lineata*, E. pavana*, E. vibilia*,	[44]	[57]	[58]
Dryadula phaetusa*	[44]	[56]	[66]
Dryas iulia*	[44]	[56]	[66]
Philaethria dido*, P. constantinoi, P. pygmalion, P. wernickei,	[44]	[56]	[66]
Agraulis vanilla*	[44]	[2]	[66]
Dione junno*, D. moneta*, D. glycera	[44]	[58]	[66]
Euptoieta claudia*	Pers. obs.	[2]	[66]
Hipparchia semele*, H. hermione, H. aristaeus, H. azorina, H. caroli, H. cretica, H. ellena, H. fagi, H. mersina, H. turcmenica	[45]	[54]	[66]
Pararge aegeria*	[39]	[19]	[66]
Morpho helenor*, M. achillaena, M. Achilles, M. menelaus, M. amphitrion, M. anaxibia, M. aurora, M. epistrophus, M. cisseis, M. cypris, M. deidamia, M. deidamia, M. hecuba, M. hercules, M. laertes, M. menelaus, M. telemachus, M. polyphemus, M. portis, M. rhetenor, M. sulkowskyi	[46]	[56]	[66]
Danaus plexippus*, D. gilippus	[47]	[55], [56]	[66]
Biblis hyperia *	[48]	[23]	[66]
Callophrys xami*, C. estela, C. guatemalena, C. johnsoni, C. millerorum, C. spinetorum	[49]	[59]	[64]
Celastrina argiolus*	Pers. obs.	[2]	[64]
Lycaena xanthoides*, L. curpreus, L. dorcas, L. editha, L. ferrisi, L. helloides, L. hermes, L. heteronea, L. ayllus, L. mariposa, L. Novalis, L. rubidus	[50]	[56]	[64]
Lemonias caliginea*	[51]	[51]	[64]
Nymphidium ariari*, N. omois*	[52]	[56], [60]	[64]

Information on signa was obtained for all taxa listed in the “Species” column, whereas data on mating pattern was obtained only for taxa marked with an asterisk. The characters used for phylogenetic reconstruction in the source phylogenies are as following: [57]: morphological and ecological; [51], [58], [59]: morphological; [60]: molecular (28S ribosomal RNA and mitochondrial ND1); [61]: molecular (mitochondrial COI-COII region and nuclear gene *wingless*); [62]: molecular (nuclear gene *wingless*). Species names were actualized according to information in www.nic.funet.fi/pub/sci/bio/life/intro.html (consulted 5/26/2011); a table with the names used in the original references can be obtained from the corresponding author.

## Results

**Figure 3:**
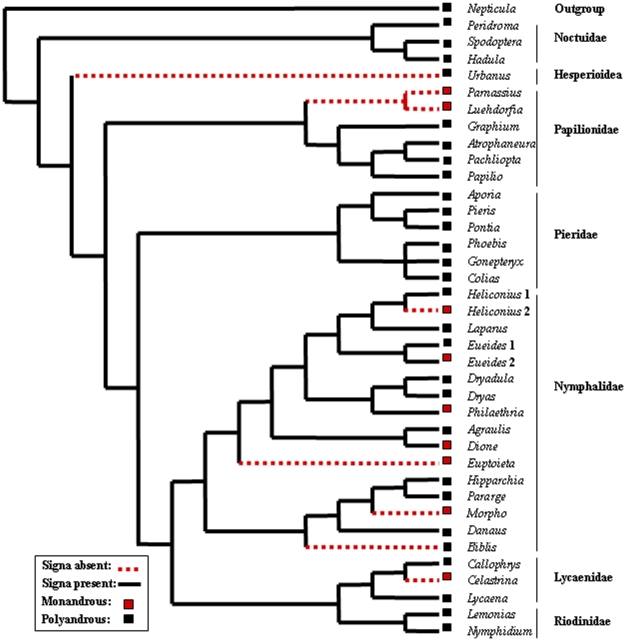
Phylogenetic mapping of mating pattern and presence of signa in a sample of Lepidoptera. Consensus supertree for 37 taxa (plus outgroup) of Lepidoptera in which female mating pattern (monandry/polyandry) and presence/absence of the female genital trait known as signa are mapped. References of source phylogenies are in Table 1.

We obtained three most parsimonious supertrees (Figure 3 shows the consensus supertree). The topology of the supertrees and the relationships between families and genera obtained are consistent with current knowledge on Lepidoptera phylogeny [30], [31].

Polyandry and presence of signa are plesiomorphic for the taxa analyzed (Figure 3). In agreement with our sexual coevolution hypothesis, only 33.3% of monandrous taxa have signa (3/9) in comparison with 93% of polyandrous taxa (27/29) (Fisher's exact probability test, *P*<0.0007). Pagel's test detected a significant association between female mating pattern and presence/absence of signa in the consensus (Figure 3) and the three most parsimonious supertrees (not shown). All tests had significant log-likelihood ratios (*df* = 4): supertree 1: 14.862 (*P*<0.005); supertree 2: 14.857 (*P*<0.01), supertree 3: 14.846 (*P*<0.01); and consensus supertree: 14.159 (*P*<0.01). Monandry evolved independently eight times and its evolution was associated with loss of signa in five cases (*Parnassius*/*Luehdorfia*, *Heliconius*, *Euptoieta*, *Morpho* and *Celastrina*). The case of *Heliconius* is illustrative: the branch that evolved monandry lost signa, whereas the branch that remained polyandrous did not. Signa were lost in seven cases, five of them (71%) in taxa that evolved monandry. Contrary to our expectations, signa were lost in two polyandrous taxa (*Urbanus* and *Biblis*) and are present in three taxa that evolved monandry independently (*Eueides*, *Philaethria* and *Dione*).

## Discussion

In general terms, our results support the hypothesis that signa evolved by sexually antagonistic coevolution. The plesiomorphy and predominance of polyandry observed were expected since polyandry prevails in insects [2], [3], [6]. As expected, most polyandrous taxa have signa and most monandrous taxa lack these structures. According to our hypothesis, when monandry evolves sperm competition disappears and selection favors thinner spermatophore envelopes because they are cheaper to produce. Thinner spermatophore envelopes are easier to break and, therefore, favor the loss of signa. In our comparative phylogenetic study, monandry evolved independently eight times and its evolution was associated with the loss of signa in five cases (62.5%).

However, the prediction that monandry favors the loss of signa also depends on the specific selective pressures responsible for the evolution of monandry. This prediction only holds if monandry is a female adaptation (i.e. when selection favors monandry in females) or if it is imposed by males via adaptations, such as genital plugs, that make thick spermatophore envelopes redundant. This second possibility could explain the evolution of monandry and loss of signa in *Parnassius*+*Luehdorfia* (Figure 3), because in these genera males produce large external mating plugs [31], known as sphragides, that block the copulatory orifice and could visually discourage male attempts to court plugged females [32]. The sphragis could have rendered the spermatophore-induced female refractory period redundant, thus favoring the evolution of the relatively small spermatophores with thin envelopes observed in these genera [33].

On the other hand, males could also impose monandry on females via the evolution of “very thick” spermatophore envelopes that still require females to use their signa to break them up—though not fast enough to permit them to remate. In this case, we expect spermatophore envelopes of monandrous species to be thicker than those of closely related polyandrous species. Our preliminary results suggest that this could be the case in *Eueides* and *Philaethria*, two of the groups in which the evolution of monandry was not associated to the loss of signa, since monandrous taxa have thicker envelopes than polyandrous taxa (Sánchez and Cordero in preparation). An alternative, and difficult to test, explanation for monandrous taxa with signa is that in these species monandry evolved recently and there has not been enough time for losing signa.

In disagreement with our hypothesis, we found two polyandrous taxa without signa (*Urbanus* and *Biblis*). Two possible explanations for these cases are that (a) in these species females evolved alternative methods for breaking spermatophore envelopes (such as chemical substances secreted within the corpus bursa), or that (b) polyandry evolved recently in these genera and there has not been enough time for (re)evolving thick envelopes and/or signa. We have no data to assess these ideas.

It is clear that a full test of our hypothesis requires information on the thickness of spermatophore envelopes, but, sadly, we haven't found any quantitative data. We are currently working on this and our preliminary data indicate that at least in *Heliconius*, as we expected, polyandrous species that posses a signum have thicker spermatophore envelopes than monandrous species lacking signum (Sánchez and Cordero in preparation). Furthermore, Matsumoto and Suzuki's [33] data on spermatophore envelope thickness and relative size of signa in Papilionidae genera differing in female mating pattern (measured by means of spermatophore counts in field collected females) agrees with our hypothesis. Envelopes are called “capsules” by these authors when they are “relatively thick” or “thick”, and an “absence” of capsule refers to a thin envelope (“capsule” interpretation kindly confirmed by Dr. Kazuma Matsumoto in an e-mail to the corresponding author dated 5/10/2004). These authors found that [33] two virtually monandrous genera (*Luehdorfia*: mean number of spermatophores ± standard error = 1.02±0.01, number of species (*n_spp_*) = 2, number of females dissected (*n_fem_*) = 98; *Parnassius*: 1.05±0.025, *n_spp_* = 2, *n_fem_* = 78) lack signa and their spermatophore envelopes are thin membranes; two slightly polyandrous genera (*Atrophaneura*: 1.18±0.06, *n_spp_* = 1, *n_fem_* = 66; *Pachliopta*: 1.2±0.2, *n_spp_* = 1, *n_fem_* = 5) have a “small signum” and “relatively thick” spermatophore envelopes; whereas two polyandrous genera (*Papilio*: 1.7±0.05, *n_spp_* = 9, *n_fem_* = 402; *Graphium*: 1.72±0.13, *n_spp_* = 2, *n_fem_* = 46) posses a “signum” and have “thick” spermatophore envelopes. Thus, female mating frequency, spermatophore envelope thickness and presence/absence of signa in this group of Papilionidae genera appear to vary in the way predicted by our sexually antagonistic coevolution hypothesis.
